# Creativity and working memory capacity in sports: working memory capacity is not a limiting factor in creative decision making amongst skilled performers

**DOI:** 10.3389/fpsyg.2015.00115

**Published:** 2015-02-10

**Authors:** Philip Furley, Daniel Memmert

**Affiliations:** Institute of Cognitive and Team/Racket Sport Research, German Sport UniversityCologne, Germany

**Keywords:** working memory, creativity, soccer, experience, divergent thinking, convergent thinking

## Abstract

The goal of the study was to investigate the relationship between domain-general working memory capacity and domain-specific creativity amongst experienced soccer players. We administered the automated operation span task in combination with a domain-specific soccer creativity task to a group of 61 experienced soccer players to address the question whether an athlete’s domain-specific creativity is restricted by their domain-general cognitive abilities (i.e., working memory capacity). Given that previous studies have either found a positive correlation, a negative correlation, or no correlation between working memory capacity and creativity, we analyzed the data in an exploratory manner by following recent recommendations to report effect-size estimations and their precision in form of 95% confidence intervals. The pattern of results provided evidence that domain-general working memory capacity is not associated with creativity in a soccer-specific creativity task. This pattern of results suggests that future research and theorizing on the role of working memory in everyday creative performance needs to distinguish between different types of creative performance while also taking the role of domain-specific experience into account.

## INTRODUCTION

The slogan of one of the most famous and successful companies in the world, Apple, is “think different.” It is not unusual that Apple’s astonishing success is attributed to the business’ policy of encouraging creativity or “thinking different,” enabling them to come up with new ways of outsmarting their competitors and opponents. Given the importance that is attributed to creativity in, for example, outsmarting one’s competitors and opponents it is not surprising that creativity has received a great deal of research attention. Recently, researchers have attempted to shed light on the underlying cognitive mechanisms associated with creative thought and behavior. In this endeavor, a recent line of research has begun to investigate the relationship between the central cognitive concept of working memory and creativity. However, the findings emerging from this line of research have been highly ambiguous, calling for further research on this topic.

Creativity can broadly be defined as the generation of ideas or problem solutions that are novel but still appropriate (Amabile, 1983; Sternberg and Lubart, 1999). Oftentimes the multifaceted term creativity is equated with the concept of divergent thinking (Guilford, 1967) in the cognitive literature which can be defined as the cognitive processes generating a broad range of solutions to a given problem (Runco, 2007). Divergent thinking is often contrasted with convergent thinking, which is defined as a deductive process that applies rules to arrive at a single, optimal solution. Besides being reported as an antithesis of divergent thinking (e.g., Guilford, 1967), convergent thinking has also been regarded as a complementary creativity process (e.g., Brophy, 2000; Dietrich, 2004; Runco, 2007). Divergent thinking is assumed to initially generate a broad range of solutions while convergent thinking discerns which solutions are the most appropriate in order to settle for the highest quality solution.

Divergent thinking has been suggested to include the cognitive measures of fluency, flexibility, and originality (Guilford, 1967). Fluency refers to the ability to generate many responses; flexibility as the ability to switch categories between responses; and originality as the ability to generate seldom responses according to the norm. In order to gain a better understanding of the cognitive underpinnings of creativity, modern creativity research (e.g., Lee and Therriault, 2013) is (re-)examining the relationship between convergent and divergent thinking and higher-order cognition (e.g., executive functions, working memory). Building on research highlighting the importance of intelligence in creative thinking (Sternberg et al., 2005; Runco, 2007), more recent endeavors have started to explore the role of working memory in creativity.

Working memory can be defined as the cognitive mechanisms capable of retaining a small amount of information in an active state for use in ongoing tasks (for reviews, see Miyake and Shah, 1999; Baddeley, 2007; Conway et al., 2007). The most important advance of the working memory model was the proposal of a system not only responsible for the storage of information but also for mechanisms of cognitive control and attention—named the central executive (Baddeley and Hitch, 1974; Baddeley, 2003). Since then, working memory has been referred to as the “blackboard of the mind” (Goldman-Rakic, 1992). It can be considered as one of the most significant achievements in human evolution as it allows to string together existing knowledge with current thoughts and ideas. According to this conceptualization, working memory intuitively seems to be an important cognitive component supporting creativity. However, the empirical evidence for this suggestion is not as clear cut as one might assume based on Goldman-Rakic (1992).

Given that two of the most important functions ascribed to working memory—keep novel information in a heightened state of activity and to discriminate between irrelevant and relevant information (Unsworth and Engle, 2007)—are also assumed to be highly relevant in creativity (De Dreu et al., 2012), it seems reasonable to assume that superior working memory functioning is associated with enhanced creativity. This positive association between measures of working memory capacity and creativity has received some empirical support.

For example, Süss et al. (2002) and Oberauer et al. (2008) demonstrated that working memory capacity was positively related to a series of different creativity tasks, involving the generation of three-word sentences, or the creation of objects out of a fixed number of elements following certain generation rules. In addition, De Dreu et al. (2012) showed that people performed worse on a creative insight task when their working memory capacity was taxed by a secondary task and that high working memory individuals showed more creative performance on divergent thinking tasks even when intelligence was controlled for. Further, they provided preliminary (as there was only an effect of working memory capacity on creative improvisations when artificially creating a creativity score over time) evidence that semiprofessional cellists performed more creative improvisations when scoring high on working memory capacity compared to cellists scoring low on working memory. Evidence along these lines was also provided by Lee and Therriault (2013) who concluded that working memory plays an important role in creative thinking because high working memory individuals are more likely to overcome interference caused by automatic, unoriginal responses, or stated differently, because high working memory individuals are better able at breaking away from a mental set or ineffective approach to a problem (see also Gilhooly et al., 2007 for a similar argumentation).

However, an increasing number of studies have also reported an opposite, negative relationship between working memory capacity and creativity (see Wiley and Jarosz, 2012, for a review). This line of research has made the argument that an important feat of working memory is to “zoom” in the focus of attention on the problem at hand, avoid distraction, and narrow the search in the problem space, and thereby, in turn, harming creative thought. Studies providing evidence that a deficit in attentional control (as measured by working memory capacity tasks, Engle, 2002, for a review) is beneficial for creative problem solving, for example, have shown that alcohol intoxication, leads to significant deficits in working memory capacity which in turn improves creative problem solving. The association between a lack of attentional control, working memory, and creativity is further supported by studies showing more creative performance amongst hyperactive children, who are characterized by working memory impairments and a decreased ability to focus their attention (Shaw, 1992; Fugate et al., 2013). Fugate et al. (2013) suggested that even amongst gifted (high IQ) children with an attention deficit hyperactivity disorder (ADHD) the relationship between working memory and a creativity index was negative, accounting for 12% of variance. Similarly, the administration of Ritalin (methylphenidate) significantly decreased symptoms of ADHD but also decreased creativity (Swartwood et al., 2003), while improving working memory capacity (Mehta et al., 2004). Evidence from brain imaging studies (Takeuchi et al., 2011) supports the line of argumentation that diffuse attention is related to individual creativity by showing that divergent thinking is positively associated with the inefficient reallocation of attention in the brain.

Given these opposing findings on the relationship between working memory and creativity, it is not surprising that other studies have failed to find any direct correlation between working memory and creativity (e.g., Takeuchi et al., 2011; Lee and Therriault, 2013). Taken together, these ambiguous findings suggest that important moderating variables influence the relationship between creative performance and working memory and have to be taken into account when investigating this relationship.

One moderating variable that has been identified to play an important role in the relationship between working memory and creativity is the type of creativity task used (Lin and Lien, 2013). With reference to dual-process theories (Evans and Stanovich, 2013, for a recent review), Lin and Lien (2013) suggested that the generation of numerous solutions, as is required in divergent thinking tasks, is more dependent on effortless, associative Type 1 processing (Evans and Stanovich, 2013) and therefore does not heavily load on working memory. According to Evans and Stanovich (2013) Type 1 processing is defined by being both initiated and completed in the presence of relevant triggering internal or external conditions. This type of processing is assumed to not require working memory. On the other hand, convergent processing as is required, for example, in creative insight tasks necessitates a more rule-based—Type 2 processing—and therefore requires working memory. Type 2 processing is usually defined as a controlled, rule-based type of processing that requires working memory for hypothetical thinking and mental simulation (Evans and Stanovich, 2013). In a series of experiments, Lin and Lien (2013) provide preliminary evidence for this suggestion.

In addition, research on the relationship between working memory and creativity in everyday settings is further complicated by the role of domain-specific knowledge in the creativity task (Wiley, 1998). In this respect, it has been suggested that expertise in a given domain facilitates problem solving by restricting attention to the most obvious solutions to the problem and suppressing less obvious options. Therefore, expertise can actually hinder creative performance in certain situations and domains by “not thinking outside the box” (Wiley and Jarosz, 2012, for a review). In an important study, participants with high levels of domain-specific knowledge and high working memory capacity were the least likely to overcome their initial mental set in order to reach a creative solution (Ricks et al., 2007).

Taken together, the existing literature on the role of working memory in creative thought and behavior highlights that this topic requires further investigation in order to gain a better understanding of the cognitive underpinnings of creativity in everyday life. The field of sport has recently been proposed to be a suitable context to investigate creative performance in a complex, ecologically valid way (Memmert, 2011). Due to the ambiguity of findings, we chose to further the understanding on the relationship between working memory and creativity by investigating this association amongst experienced soccer players within their field of experience. In particular, we were interested in the question of whether an athlete’s domain-specific creativity might be restricted by their domain-general cognitive abilities (i.e., working memory capacity). In order to address this question, we administered a domain-general measure of working memory capacity (the automated operation span, Unsworth et al., 2005) in combination with a domain-specific sport creativity task (Memmert et al., 2013).

The rationale for using the automated operation span task was derived from the controlled attention theory of working memory capacity (Engle, 2002, for a review) which suggests that domain-general measures of working memory capacity predict higher order cognition such as, e.g., language comprehension (King and Just, 1991) or reasoning (Kyllonen and Christal, 1990), because of the domain general controlled attention component shared by these tasks and the working memory capacity tasks. Consistent with this view, a modification of the reading span task that requires mathematical processing instead of comprehending sentences is still an excellent predictor of language comprehension (e.g., Engle, 2002). In working memory capacity measures participants generally have to memorize digits or words while solving a demanding, secondary processing task such as verifying equations. In this respect, these tasks measure the ability of individuals to keep task-relevant information in a state of heightened activity during the execution of a processing task. Hence, the automated operation span task is a well-suited domain-general measure that has proven to be suitable to predict domain-specific performance (e.g., Furley and Memmert, 2012). This study demonstrated that ice hockey players with a low working memory capacity failed to adjust their tactical decisions to the demands of the game situation and more often “blindly” followed a tactical instruction they got from the coach during a simulated time-out, even though it was not appropriate for the game situation. Importantly, ice hockey players with a high working memory capacity were more proficient at adjusting their tactical decision to the demands of the situation instead of relying on the information they got during a simulated team time-out that was not appropriate for the following offensive game situation. No differences between high and low working memory capacity ice-hockey players were evident in situations in which the tactical information they got in the team time-out was helpful for the following game situation as there was no inner conflict between possible solutions to be resolved, and therefore the situation did not require attentional control.

The rationale for choosing the creativity task of Memmert et al. (2013) was that this task paradigm has been shown to have good psychometric properties for measuring both divergent (Johnson and Raab, 2003; Memmert, 2010a) and convergent thinking (Memmert, 2010b). The chosen criteria for creative solutions in team sport (originality, flexibility, and fluency) have been derived from the state-of-the-art creativity research (Sternberg, 1999; Runco, 2007; Antonietti et al., 2013) and have successfully been transferred to the context of sports in numerous studies (for a review Memmert, 2013, 2015a; also Memmert and Roth, 2007; Memmert and Perl, 2009a,b; Memmert, 2010b).

In the present study we test the hypothesis whether domain-general working memory capacity is a restricting factor in the creativity of soccer players. Given the outlined controversial findings on the relationship between working memory capacity and creativity, we test this two-sided hypothesis by conducting both null-hypothesis significance tests, while also following recent recommendations (Cumming, 2012, 2014) of reporting effect-size estimations and their precision in form of 95% confidence intervals.

## MATERIALS AND METHODS

### PARTICIPANTS

Sixty one male soccer athletes (*M*_age_ = 23.48, SD = 3.6) took part in the study. Their average playing experience was 17.6 years (SD = 3.9) at an amateur to semi-professional level in Germany. The athletes reported to spend an average of 5.7 h/week (SD = 4.4) of playing or training soccer. None of these variables significantly influenced the pattern of results. Written informed consent was obtained from every participant before commencing the experiment. The study was carried out in accordance with the Helsinki Declaration of 1975.

### EXPERIMENTAL TASK AND MEASURES

#### Working Memory measure

We used the well-established automated operation span score as an index of working memory capacity (Unsworth et al., 2005). As in the original operation span task (Turner and Engle, 1989) participants had to solve math problems while trying to remember an unrelated set of letters. The task included a total of 15 trials (three trials each with 3, 4, 5, 6, and 7 letters to remember). An example of a three-item trial might be: is (8/2) – 1 = 1? (correct/incorrect?) →*F*; is (6 ^∗^ 1) + 2 = 8? (correct/incorrect?) →*P*; is (10 ^∗^ 2) - 5 = 15? (correct/incorrect?) →*Q*. After verifying the three equations in this example, participants were asked to select the presented letters with a mouse click from an array of 12 potential letters in the order they were presented (in this case *F, P, Q*). The primary measure of working memory capacity was the Ospan score (Unsworth et al., 2005), calculated as the total number of letters recalled across all error-free trials. See Unsworth et al. (2005) for full task details. The task lasted approximately 15 min.

#### Creativity task

We adapted the soccer-specific divergent-thinking test (see Memmert et al., 2013, for full details) consisting of 20 different video clips displaying offensive soccer scenes that allowed for a variety of possible solutions when the video stopped with one offensive player in possession of the ball. The test was created in assistance with two independent soccer experts in possession of high-level trainer certifications from a large battery of soccer matches from 2010/2011. The final 20 scenes that comprised the soccer-specific creativity test (Memmert et al., 2013) were those for which the experts had agreed upon offering the most tactical decision options. Each scene was approximately 10 s long, after which it was stopped and the last frame was shown for an additional 3.5 s before it faded away to a black screen. This frame showed an attacking player in possession of the ball, with a variety of tactical options to his disposal.

### PROCEDURE

Participants were recruited from local football clubs and tested individually in a quite laboratory on a standard 15 inch notebook. After filling out a questionnaire, gathering biographic data, participants were randomly allocated to either first take the automated operation span or the soccer-specific divergent thinking test to avoid potential order effects. Altogether, testing took approximately 50 min. E-prime 2.0 professional (Psychological Software Tools, 2007) was used to administer both the automated operation span task and the soccer-specific divergent thinking task. The instructions were standardized and presented on the computer screen. For the divergent thinking task, participants were instructed to assume the role of the player in possession of the ball. Half of the participants viewed 10 videos and 10 stills presented in random order, while for the other group this was reversed and the 10 videos were presented as stills and the 10 stills as videos. The rationale for this was to explore the difference between dynamic and static information in domain-specific creative problem-solving as dynamic information is more representative of the decision making demands experienced soccer-players are confronted with in their performance environments (Helsen and Starkes, 1999; Williams and Ericsson, 2005). As no differences were evident between static and dynamic scenes we collapsed data analysis over both categories. After every stimulus presentation participants had to write down all the tactical decision making options that came to their mind. Participants had 45 s time (the time was indicated by a countdown after every stimulus presentation on the screen) to generate as many adequate tactical solutions as possible (divergent thinking) and then bring these generated options in a hierarchical order (within the 45 s time frame) with option one being the option that they would actually decide upon in that situation (convergent thinking). After completing the testing procedure, participants were informed about the purpose of the experiment.

### DATA ANALYSIS

Soccer-specific divergent thinking was assessed by using the three criteria of fluency, flexibility, and originality (see Guilford, 1967; Runco, 2007). Fluency was simply assessed by the number of tactical solutions produced by a participant. Flexibility was measured via diversity of responses. All solutions given by the participants were sorted into seven different categories based on Memmert et al. (2013: shot on goal, feint followed by a pass, dribble, short pass, lob, cross, and miscellaneous). One point was given for each category selected by a subject and summed for the respective stimulus, before being divided by the total number of stimuli to arrive at a flexibility score for every participant. Two independent raters (soccer experts with high-level coaching certifications) judged the originality of the solutions for each scene. The soccer experts were not familiar with any other variables about the participants. The available range for the originality assessments was 1 (not original at all) to 5 (very original). The inter-judge reliability coefficient was above the critical limit of 0.80 (intraclass correlation coefficient). The individual ratings of the stimuli were used to compute a mean originality score for each participant (the ratings from both raters were averaged for every stimulus and then summed up before being divided by the total number of responses). Besides analyzing the three components of divergent thinking, we further computed a creativity value by averaging the *z*-transformed fluency, flexibility, and originality values.

Further, the same two soccer experts who rated the originality of the responses agreed upon an optimal solution for every scene which served as an index for the best solution participants could have chosen. As a measure for convergent thinking we compared the correspondence of participants’ ratings with the experts’ best solution and summed up the number of correspondences before dividing them by the total number of scenes.

We analyzed the relationship between working memory capacity and the measures of creativity by computing Pearson’s correlation coefficients and corresponding confidence intervals. Further we compared the upper and lower working memory quartiles with a series of independent *t*-tests (all two-tailed).

## RESULTS

**FIGURE 1:**
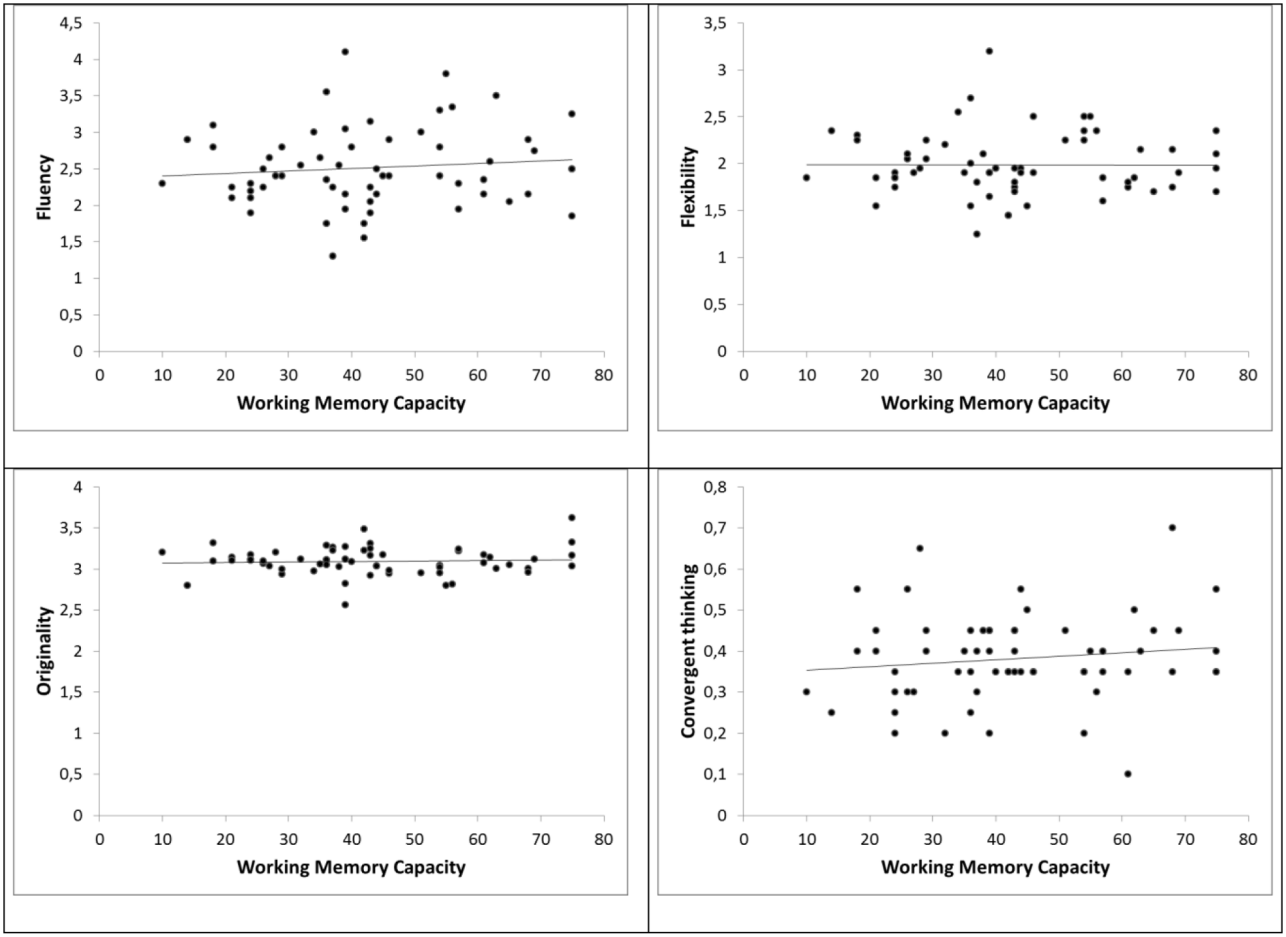
**Bivariate correlations between working memory capacity and the three divergent thinking measures (fluency, flexibility, and originality) and working memory capacity with the convergent thinking measure**.

Pearson’s correlation coefficients for the operation span and the different measures of creativity are shown in **Table 1** and their graphical equivalent in **Figure 1**. The pattern of results clearly shows no relationship between domain-general working memory capacity and domain-specific creativity. Even when only comparing the 25% highest (*M =* 65.7, SD = 7.1) and 25% lowest (*M* = 23.5, SD = 4.4; *t*(28) = - 19.649*; p* < 0.001, *d* = 7.1) working memory capacity athletes—which is common practice in the working memory capacity literature (Engle, 2002, for a review)—no significant differences emerged for the combined creativity value (*t*(28) = - 0.560; *p* = 0.58, *d* = 0.204), the fluency value [*t*(28) = - 0.752*; p* = 0.46, *d* = 0.275], the flexibility value [*t*(28) = 0.641*; p* = 0.53, *d* = 0.233], and the originality value [*t*(28) = - 0.749*; p* = 0.46, *d* = 0.273].

**Table 1:** Correlations (Pearson’s *r*) coefficients for working memory capacity and the creativity measures.

	WMC	Divergent	Fluency	Flexibility	Originality	Convergent
WMC	-	0.102	0.107	-0.004	0.061	0.132
Divergent	[-0.15,0.35]	-	0.835**	-0.821**	-0.051	0.056
Fluency	[-0.15,0.35]	[0.74,0.89]	-	-0.868**	-0.530**	0.105
Flexibility	[-0.26,0.25]	[0.72,0.89]	[0.79,0.92]	-	-0.552**	-0.034
Originality	[-0.19,0.31]	[-0.30,0.20]	[-0.69,-0.32]	[-0.71,-0.35]	-	0.019
Convergent	[-0.12,0.37]	[-0.20,0.31]	[-0.15,-0.35]	[-0.28,0.22]	[-0.23,0.27]	-

The lower and upper bounds of the 95% confidence interval are shown in square brackets in the bottom left half of the table. Correlations that are significant (α = 0.01; two-tailed) are marked with **.

Further, the correlation between working memory capacity and a measure of convergent thinking—the final option chosen—was not significant (cf. **Table 1**), indicating that a high domain-general working memory capacity is not associated with better decisions in soccer. This was also evident when comparing the 25% highest and 25% lowest working memory capacity athletes [*t*(28) = - 0.429*; p* = 0.67, *d* = 0.156]. This finding is in line with Furley and Memmert (2012) who provided evidence that a higher working memory capacity is only associated with superior decision making in certain situations, e.g., when a predominant response tendency interferes with the best solution in a situation or when there is external distraction from the decision making task. However, there was no association between overall decision quality and working memory capacity.

## DISCUSSION

The aim of this study was to explore the relationship between domain-general working memory capacity and domain-specific creativity amongst experienced soccer players. The pattern of results provides evidence that domain-general working memory capacity was not associated with creativity in a soccer-specific creativity task. Thus, our findings do not support the previously reported suggestion of a positive relationship between a domain-general measure of working memory capacity and domain-specific creativity (De Dreu et al., 2012). The present findings are in line with existing studies that do not find any direct correlation between working memory and creativity (e.g., Takeuchi et al., 2011; Lee and Therriault, 2013). Therefore, our results suggest that the moderating role of the nature of the creativity task plays an important role in the interaction between divergent thinking and working memory, as it is evident in current creativity research (for reviews, see Kasof, 1997). Or as Fugate et al. (2013, p. 236) pointed out: “In sum, the mediating effect of working memory on creativity depends on the type of task to be performed.” In this respect, the present findings are well aligned with current theorizing (see Wiley and Jarosz, 2012, for a review) on the role of working memory capacity in problem solving, concluding that successful problem solving depends on the needs of the situation.

While an increasing number of correlational studies and laboratory-based experiments have started investigating creativity and working memory, there are only few studies which take task complexity and domain-specific knowledge in regard to the task into consideration. The present research provides a first attempt of filling this gap in the literature. However, the present research is not without limitations. Although, we provide evidence that domain-general working memory capacity was not related with domain-specific creativity amongst experienced soccer players, we did not experimentally manipulate domain-specific experience by either varying the task demands or the experience level of the participants. As we were interested in answering the question whether an athlete’s domain-specific creativity is restricted by their domain-general cognitive abilities (i.e., working memory capacity), it is currently not clear whether less experienced athletes or children would have benefitted on the creativity task from having a greater working memory capacity. Further in consideration of the findings of Ricks et al. (2007) who showed that expertise in combination with high working memory capacity can hinder creative performance, top-level soccer players (as compared to the amateur to semi-professional participants) might have been influenced by their working memory capacity on the creativity task. Therefore, future research and theorizing on the role of working memory in creative behavior needs to distinguish between different types of creative performance while considering the role of domain-specific experience in the creativity task. A fruitful approach in this endeavor would be to manipulate task demands (requiring domain-specific knowledge or not) while having various participant groups varying in domain-specific experience and working memory capacity.

Given the importance of creative moments, products, and processes in a variety of contexts, such as economy, medicine, science, or sports, the present research contributes to a growing body of literature that sheds light on the underlying cognitive mechanisms associated with creative thought and behavior. Specifically, we demonstrated that working memory capacity was not a limiting factor on creative decision making amongst skilled performers. Therefore, experienced soccer players did not benefit from a superior working memory capacity in finding creative solutions to soccer-specific situations. However, similar to previous research in psychology showing that a narrow focus of attention is detrimental to creativity (Wiley and Jarosz, 2012, for a review), studies in the context of sports have demonstrated impaired creative problem solving by narrowing the focus of attention via specific instructions amongst children (Memmert, 2007; Memmert and Furley, 2007) and adult athletes (Furley et al., 2010; Memmert, 2015b). This might suggest that although individual differences in focused attention (as measures by working memory capacity, Engle, 2002) did not contribute to creativity, situational manipulations of available working memory capacity (e.g., taxing working memory by a secondary task, cf. De Dreu et al., 2012, study 1) might affect creative problem solving. Future research might want to look into this possibility.

## AUTHOR CONTRIBUTIONS

PF and DM developed the study concept, and both authors contributed to the design. PF collected the data and analyzed it in collaboration with DM. PF wrote the first draft of the manuscript, and DM helped edit and revise it. Both authors approved the final, submitted version of the manuscript.

## Conflict of Interest Statement

The authors declare that the research was conducted in the absence of any commercial or financial relationships that could be construed as a potential conflict of interest.

## References

[B1] Amabile T. M. (1983). The social psychology of creativity: a componential conceptualization. *J. Pers. Soc. Psychol.* 45 357–376 10.1007/978-1-4612-5533-8

[B2] Antonietti A. Colombo B. Memmert D. (2013). *Psychology of Creativity: Advances in Theory, Research and Application*. Hauppauge, NY: Nova Science Publishers.

[B3] Baddeley A. D. (2003). Working memory: looking back and looking forward. *Nat. Rev. Neurosci.* 4 829–839 10.1038/nrn1201 14523382

[B4] Baddeley A. D. (2007). *Working Memory, Thought, and Action*. Oxford: Oxford University Press 10.1093/acprof:oso/9780198528012.001.0001

[B5] Baddeley A. D. Hitch G. J. (1974). “Working memory,” in *The Psychology of Learning and Motivation: Advances in Research and Theory*, Vol. 8 ed.Bower G. H. (New York: Academic Press), 47–89 10.1016/S0079-7421(08)60452-1

[B6] Brophy D. R. (2000). Comparing the attributes, activities, and performance of divergent, convergent, and combination thinkers. *Creat. Res. J.* 13 439–455 10.1207/S15326934CRJ1334-20

[B7] Conway A. R. A. Jarrold C. Kane M. J. Miyake A. Towse J. N. (2007). *Variation in Working Memory.* New York: Oxford University Press.

[B8] Cumming G. (2012). *Understanding the new statistics. Effect Sizes, Confidence Intervals, and Meta-Analysis*. New York: Routledge.

[B9] Cumming G. (2014). The new statistics: why and how. *Psychol. Sci.* 25 7–29 10.1177/0956797613504966 24220629

[B10] De Dreu C. K. Nijstad B. A. Baas M. Wolsink I. Roskes M. (2012). Working memory benefits creative insight, musical improvisation, and original ideation through maintained task-focused attention. *Pers. Soc. Psychol. Bull.* 38 656–669 10.1177/0146167211435795 22301457

[B11] Dietrich A. (2004). The cognitive neuroscience of creativity. *Psychon. Bull. Rev.* 11 1011–1026 10.3758/BF03196731 15875970

[B12] Engle R. W. (2002). Working memory capacity as executive attention. *Curr. Dir. Psychol. Sci.* 11 19–23 10.1111/1467-8721.00160

[B13] Evans J. St. B. T. Stanovich K. E. (2013). Dual-process theories of higher cognition: advancing the debate. *Perspect. Psychol. Sci.* 8 223–241 10.1177/1745691612460685 26172965

[B14] Fugate C. M. Zentall S. S. Gentry M. (2013). Creativity and working memory in gifted students with and without characteristics of attention deficit hyperactive disorder: lifting the mask. *Gift. Child Q.* 57 234–246 10.1177/0016986213500069

[B15] Furley P. Memmert D. (2012). Working memory capacity as controlled attention in tactical decision making. *J. Sport Exerc. Psychol.* 34 322–344 10.1371/journal.pone.0062278 22691397

[B16] Furley P. Memmert D. Heller C. (2010). The dark side of visual awareness in sport – inattentional blindness in a real-world basketball task. *Atten. Percept. Psychophys.* 72 1327–1337 10.3758/APP.72.5.1327 20601714

[B17] Gilhooly K. J. Fioratou E. Anthony S. H. Wynn V. (2007). Divergent thinking: strategies and executive involvement in generating novel uses for familiar objects. *Br. J. Psychol.* 98 611–625 10.1111/j.2044-8295.2007.tb00467.x 17535464

[B18] Goldman-Rakic P. S. (1992). Working memory and the mind. *Sci. Am.* 267 111–117 10.1038/scientificamerican0992-110 1502513

[B19] Guilford J. P. (1967). *The Nature of Human Intelligence.* New York: McGraw-Hill.

[B20] Helsen W. F. Starkes J. L. (1999). A multidimensional approach to skilled perception and performance in sport. *Appl. Cogn. Psychol.* 13 1–27 10.1002/(SICI)1099-0720(199902)13

[B21] Johnson J. G. Raab M. (2003). ‘Take the first’: option-generation and resulting choices. *Organ. Behav. Hum. Decis. Process.* 91 215–229 10.1016/S0749-5978(03)00027-X

[B22] Kasof J. (1997). Creativity and breadth of attention. *Creat. Res. J.* 10 303–315 10.1207/s15326934crj1004-2

[B23] King J. Just M. A. (1991). Individual differences in syntactic processing: the role of working memory. *J. Mem. Lang.* 30 580–602 10.1016/0749-596X(91)90027-H

[B24] Kyllonen P. C. Christal R. E. (1990). Reasoning ability is (little more than) working-memory capacity?! *Intelligence* 14 389–433 10.1016/S0160-2896(05)80012-1

[B25] Lee C. S. Therriault D. J. (2013). The cognitive underpinnings of creative thought: a latent variable analysis exploring the roles of intelligence and working memory in three creative thinking processes. *Intelligence* 41 306–320 10.1016/j.intell.2013.04.008

[B26] Lin W. L. Lien Y. W. (2013). The different role of working memory in open-ended versus closed-ended creative problem solving: a dual-process theory account. *Creat. Res. J.* 25 85–96 10.1080/10400419.2013.752249

[B27] Mehta M. A. Goodyer I. M. Sahakian B. J. (2004). Methylphenidate improves working memory and set-shifting in AD/HD: relationships to baseline memory capacity. *J. Child Psychol. Psychiatry* 45 293–305 10.1111/j.1469-7610.2004.00221.x 14982243

[B28] Memmert D. (2007). Can creativity be improved by an attention-broadening training program? – An exploratory study focusing on team sports. *Creat. Res. J.* 19 281–292 10.1080/10400410701397420

[B29] Memmert D. (2010a). Creativity, expertise, and attention: exploring their development and their relationships. *J. Sports Sci.* 29 93–104 10.1080/02640414.2010.528014 21104518

[B30] Memmert D. (2010b). Testing of tactical performance in youth elite soccer. *J. Sports Sci. Med.* 9 199–205. 24149686 PMC3761738

[B31] Memmert D. (2011). “Sports and creativity,” in *Encyclopedia of Creativity*, 2nd Edn, Vol. 2 eds Runco M. A. Pritzker S. R. (San Diego: Academic Press), 373–378 10.1016/B978-0-12-375038-9.00207-7

[B32] Memmert D. (2013). “Tactical creativity,” in *Routledge Handbook of Sports Performance Analysis*, eds McGarry T. O’Donoghue P. Sampaio J. (Abingdon: Routledge), 297–308.

[B33] Memmert D. (2015a). *Teaching Tactical Creativity in Team and Racket Sports: Research and Practice*. Abingdon: Routledge.

[B34] Memmert D. (2015b, forthcoming). “Attention in sports,” in *The Handbook of Attention*, eds Fawcett J. Risko E. F. Kingstone A. (Cambridge: MIT Press).

[B35] Memmert D. Furley P. (2007). “I spy with my little eye!” – breadth of attention, inattentional blindness, and tactical decision making in team sports. *J. Sport Exerc. Psychol.* 29 365–381. 17876972 10.1123/jsep.29.3.365

[B36] Memmert D. Hüttermann S. Orliczek J. (2013). Decide like lionel messi! The impact of regulatory focus on divergent thinking in sports. *J. Appl. Soc. Psychol.* 43 2163–2167 10.1111/jasp.12159

[B37] Memmert D. Perl J. (2009a). Game creativity analysis by means of neural networks. *J. Sports Sci.* 27 139–149 10.1080/02640410802442007 19058086

[B38] Memmert D. Perl J. (2009b). Analysis and simulation of creativity learning by means of artificial neural networks. *Hum. Mov. Sci.,* 28 263–282 10.1016/j.humov.2008.07.006 19110331

[B39] Memmert D. Roth K. (2007). The effects of non-specific and specific concepts on tactical creativity in team ball sports. *Sports Sci.* 25 1423–1432 10.1080/02640410601129755 17786695

[B40] Miyake A. Shah P. (1999). *Models of Working Memory: Mechanisms of Active Maintenance and Executive Control*. Cambridge: Cambridge University Press 10.1017/CBO9781139174909

[B41] Oberauer K. Süss H.-M. Wilhelm O. Wittmann W. (2008). Which working memory functions predict intelligence? *Intelligence* 36 641–652 10.1016/j.intell.2008.01.007

[B42] Psychological Software Tools (2007). *E-Prime [Computer Software].* Pittsburgh, PA: Psychological Software Tools.

[B43] Ricks T. R. Turley-Ames K. J. Wiley J. (2007). Effects of working memory capacity on mental set due to domain knowledge. *Mem. Cogn.* 35 1456–1462 10.3758/BF03193615 18035641

[B44] Runco M. A. (2007). *Creativity: Theories and Themes: Research, Development, and Practice*. San Diego, CA: Academic Press.

[B45] Shaw G. A. (1992). Hyperactivity and creativity: the tacit dimension. *Bull. Psychon. Soc.* 30 157–160 10.3758/BF03330426

[B46] Sternberg R. J. (ed.). (1999). *Handbook of Creativity.* New York, NY: Cambridge University Press.

[B47] Sternberg R. J. Lubart T. I. (1999). “The concept of creativity: prospects and paradigms,” in *Handbook of Creativity*, ed.Sternberg R. J. (New York, NY: Cambridge University Press), 3–15.

[B48] Sternberg R. J. Lubart T. I. Kaufman J. C. Pretz J. E. (2005). “Creativity,” in *The Cambridge Handbook of Thinking and Reasoning*, eds Holyoak K. J. Morrison R. G. (Cambridge, MA: Cambridge University Press), 351–369.

[B49] Süss H.-M. Oberauer K. Wittmann W. W. Wilhelm O. Schulze R. (2002). Working-memory capacity explains reasoning ability—and a little bit more. *Intelligence* 30 261–288 10.1016/S0160-2896(01)00100-3

[B50] Swartwood M. O. Swartwood J. N. Farrell J. (2003). Stimulant treatment of ADHD: effects of creativity and flexibility in problem solving. *Creat. Res. J.* 15 417–419 10.1207/S15326934CRJ1504-9

[B51] Takeuchi H. Taki Y. Hashizume H. Sassa Y. Nagase T. Nouchi R. (2011). Failing to deactivate: the association between brain activity during a working memory task and creativity. *Neuroimage* 55 681–687 10.1016/j.neuroimage.2010.11.052 21111830

[B52] Turner M. L. Engle R. W. (1989). Is working memory capacity task dependent? *J. Mem. Lang.* 28 127–154 10.1016/0749-596X(89)90040-5

[B53] Unsworth N. Engle R. W. (2007). The nature of individual differences in working memory capacity: active maintenance in primary memory and controlled search from secondary memory. *Psychol. Rev.* 114 104–132 10.1037/0033-295X.114.1.104 17227183

[B54] Unsworth N. Heitz R. P. Schrock J. C. Engle R. W. (2005). An automated version of the operation span task. *Behav. Res. Methods* 37 498–505 10.3758/BF03192720 16405146

[B55] Wiley J. (1998). Expertise as mental set: the effects of domain knowledge in creative problem solving. *Mem. Cogn.* 26 716–730 10.3758/BF03211392 9701964

[B56] Wiley J. Jarosz A. F. (2012). Working memory capacity, attentional focus, and problem solving. *Curr. Dir. Psychol. Sci.* 21 258–262 10.1177/0963721412447622

[B57] Williams A. M. Ericsson K. A. (2005). Perceptual-cognitive expertise in sport: some considerations when applying the expert performance approach. *Hum. Mov. Sci.* 24 283–307 10.1016/j.humov.2005.06.002 16095739

